# Comparing qPCR and DNA methylation-based measurements of telomere length in a high-risk pediatric cohort

**DOI:** 10.18632/aging.203849

**Published:** 2022-01-24

**Authors:** Waylon J. Hastings, Laura Etzel, Christine M. Heim, Jennie G. Noll, Emma J. Rose, Hannah M.C. Schreier, Chad E. Shenk, Xin Tang, Idan Shalev

**Affiliations:** 1 Department of Biobehavioral Health, The Pennsylvania State University, University Park, PA 16802, USA; 2 Charité – Universitätsmedizin Berlin, Corporate Member of Freie Universität Berlin and Humboldt Universität zu Berlin, Institute of Medical Psychology, Berlin, Germany; 3 Department of Human Development and Family Studies, The Pennsylvania State University, University Park, PA 16802, USA; 4 The Edna Bennett Pierce Prevention Research Center, The Pennsylvania State University, University Park, PA 16802, USA; 5 Department of Pediatrics, The Pennsylvania State University College of Medicine, Hershey, PA 17033, USA

**Keywords:** telomere length, DNAmTL, qPCR, pediatric, agreement

## Abstract

Various approaches exist to assess population differences in biological aging. Telomere length (TL) is one such measure, and is associated with disease, disability and early mortality. Yet, issues surrounding precision and reproducibility are a concern for TL measurement. An alternative method to estimate TL using DNA methylation (DNAmTL) was recently developed. Although DNAmTL has been characterized in adult and elderly cohorts, its utility in pediatric populations remains unknown. We examined the comparability of leukocyte TL measurements generated using qPCR (absolute TL; aTL) to those estimated using DNAmTL in a high-risk pediatric cohort (*N* = 269; age: 8–13 years, 83% investigated for maltreatment). aTL and DNAmTL measurements were correlated with one another (r = 0.20, *p* = 0.001), but exhibited poor measurement agreement and were significantly different in paired-sample *t*-tests (Cohen’s d = 0.77, *p* < 0.001). Shorter DNAmTL was associated with older age (r = −0.25, *p* < 0.001), male sex (β = −0.27, *p* = 0.029), and White race (β = −0.74, *p* = 0.008). By contrast, aTL was less strongly associated with age (r = −0.13, *p* = 0.040), was longer in males (β = 0.31, *p* = 0.012), and was not associated with race (*p* = 0.820). These findings highlight strengths and limitations of high-throughput measures of TL; although DNAmTL replicated hypothesized associations, aTL measurements were positively skewed and did not replicate associations with external validity measures. These results also extend previous research in adults and suggest that DNAmTL is a sensitive TL measure for use in pediatric populations.

## INTRODUCTION

Telomeres are repetitive nucleoprotein regions at chromosome ends that prevent end to end fusions and maintain chromosome stability [1]. Telomeres incrementally shorten each time a cell divides, leading to age-associated decreases in telomere length (TL) across the lifespan [2]. Large population studies have associated shorter TL with a range of health problems including cardiovascular disease, high blood pressure, cancer, and shorter life expectancy [3–5]. In addition, studies have shown that TL can be modulated by risk factors that are associated with shorter life expectancy, including substance abuse [6], poverty [7], and early-life adversity [8]. For these reasons, telomere attrition is considered a hallmark of biological aging [9]. Despite a plethora of literature relating TL to biological aging and disease processes [10], methodological and inferential challenges associated with their use have led to concerns about the validity of TL as a biomarker of aging [11].

Several methods for measuring TL have been developed, each with unique strengths and limitations [12]. Measurement of TL using Southern blot is widely regarded as a reliable method, providing an objective measure of average TL per chromatid end in kilobases (kb) with high precision and reproducibility [13]. However, the Southern blot method is difficult to implement in epidemiological studies due to its reliance on high-quality concentrated DNA, the technical expertise needed to perform the assay, and limited throughput [14]. The most common method to quantify TL in epidemiological research is quantitative-PCR (qPCR), which expresses telomeric content (T) relative to a single-copy gene (S) via a unitless metric known as the T/S ratio [15]. This technique requires substantially less DNA than Southern blot and is high-throughput, capable of analyzing over 100 samples on a single assay. Several iterations of the qPCR method have been introduced since its original development, including means of simultaneously estimating telomeric content and single-copy gene within the same well [16], as well as methods incorporating an oligomer standard-curve to approximate absolute TL (aTL) in kb [17].

Although widely used, concerns about the precision of TL measurement via qPCR remain. Several factors can influence qPCR precision, including DNA extraction method, sample storage conditions, and PCR mastermix, to name a few [18–20]. In one international collaborative study, the coefficient of variation (CV) across replicate qPCR measurements ranged from 2.34% to 34.15%, with an average of 18.31% [21]. Diminished assay precision makes it difficult to disentangle between-group differences from measurement error. For example, cross-sectional studies using the Southern blot method report between-group differences of a few dozen to several hundred base-pairs depending on the outcome tested [22–24]. With a lifespan range of TL near 12.5 kb to 4.5 kb [25, 26], a low qPCR assay CV of 2% translates to measurement error of 90 bp-250 bp, possibly obscuring meaningful differences between groups. Measurement error of this sort, combined with insufficient follow-up duration, may also contribute to the telomere lengthening conundrum sometimes observed in longitudinal studies [27, 28], a finding counter to studies using larger samples or Southern Blot techniques that report decreases in TL on the scale of 20 bp-60 bp per year [2, 29–31].

In response to ongoing concerns about the reliability of TL measurement via qPCR, alternative approaches have emerged, including a DNA methylation-based estimator of telomere length (DNAmTL) [32]. Although initially developed to predict TL measured by Southern Blot, DNAmTL exhibited superior mortality prediction and stronger associations with cardiovascular outcomes than TL measurements generated using Southern Blot [32]. Moreover, exceptionally long-lived individuals exhibited longer DNAmTL in the absence of such differences using TL measured via qPCR [33]. While DNAmTL has been validated using adult and elderly samples [34], its performance within pediatric cohorts remains unknown.

To address these gaps, we explored associations between TL measurements generated using both qPCR (aTL) and DNA methylation (DNAmTL) in a subset of participants from the ongoing Child Health Study (CHS). The CHS is a large multidisciplinary study designed to provide prospective, longitudinal data on the health and development of children with and without a history of maltreatment to better inform intervention research and reveal opportunities for reversibility [35]. We leveraged existing data from Time 1 (baseline) of the CHS to conduct cross-sectional analyses on the performance of each TL measure in relation to known metrics of external validity, as recommended for comparative studies [36]. Specifically, we examined each measure’s ability to capture differences as a function of age, sex, race, and ethnicity [2, 37, 38], as well as each measure’s responsiveness to early adversity, a life course exposure linked to accelerated biological aging and telomere attrition [8]. We also conducted exploratory analyses investigating associations with metrics less validated in pediatric populations, including paternal age effects [39] and concordance with pubertal development [40]. We predicted that both shorter aTL and shorter DNAmTL will be associated with older age, male sex, White race, exposure to maltreatment, advanced pubertal development and younger paternal age.

## RESULTS

### Sample demographics

Demographics for the analytical sample and distinguished by maltreatment versus comparison youth are shown in Table 1. No significant differences were observed in mean chronological age or distribution of sex, race, and ethnicity between the maltreatment and comparison groups. The maltreatment group did exhibit significantly higher BMI, younger paternal age at birth, lower family income, and more advanced pubertal development.

**Table 1:** Demographics for the analytical sample distinguished by investigation for maltreatment exposure.

	**Full Sample (*N* = 269)**	**Comparison (*N* = 47)**	**Maltreatment (*N* = 222)**	***p*-value**
	**Mean/% (SD)**	**Mean/% (SD)**	**Mean/% (SD)**	
Age (years)	11.38 (1.47)	11.13 (1.49)	11.43 (1.47)	0.210
BMI	21.78 (6.02)	20.24 (5.30)	22.10 (6.12)	**0.037**
Income ($10,000/year)	3.75 (3.47)	5.77 (3.95)	3.33 (3.22)	**<0.001**
Tanner Stage	2.44 (1.05)	2.06 (1.01)	2.52 (1.04)	**0.007**
Paternal Age at Birth (years)	29.32 (7.80)	31.54 (7.06)	28.81 (7.89)	**0.031**
Sex				
Male	48.7%	48.9%	48.6%	0.999
Female	51.3%	51.1%	51.4%	
Race				
White	68.4%	80.9%	65.8%	0.053
Black/African American	16.4%	14.9%	16.7%	
Other	15.2%	4.3%	17.6%	
Ethnicity				
Hispanic	11.5%	4.3%	13.1%	0.143
Non-Hispanic	88.5%	95.7%	86.9%	
DNAmTL (kb)	8.04 (0.18)	8.07 (0.16)	8.03 (0.19)	0.191
aTL (kb)	9.88 (3.24)	9.51 (3.39)	9.95 (3.21)	0.415

### Concordance among TL measures and age-associated change in TL

**Figure 1:**
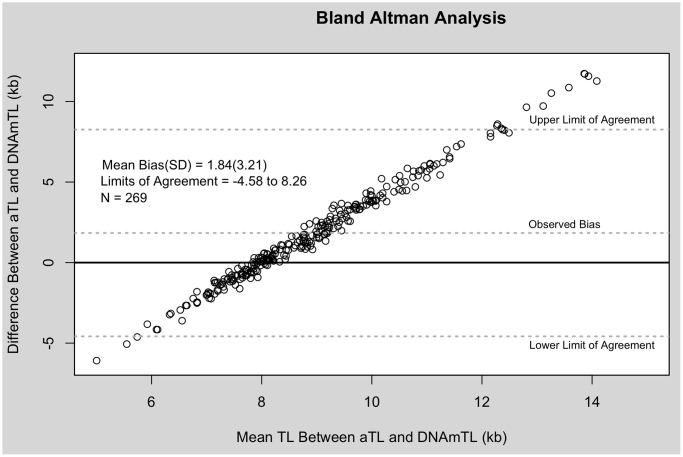
**Bland Altman analysis of aTL and DNAmTL.** X-axis represents the average of the two measures. The Y-axis represents the difference between the two measures. Each point corresponds to one paired comparison.

TL measurements estimated using qPCR were significantly longer than those estimated using DNA methylation in paired sample *t*-tests (Cohen’s d = 0.77, *p* < 0.001). Bland Altman analysis revealed a mean bias of 1.84 kb and wide limit of agreement (−4.58 to 8.26 kb). DNAmTL measurements fell within a narrower window, whereas aTL tended to overestimate the longest telomeres (Figure 1).

**Figure 2:**
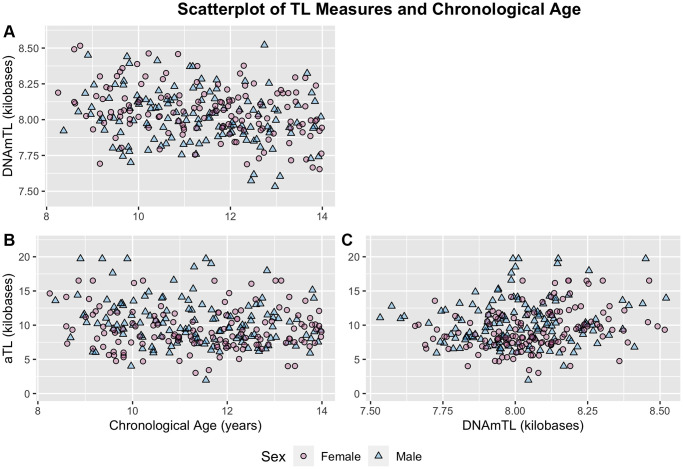
**Scatterplots of chronological age and TL measures distinguished by sex.** (**A**) DNAmTL and chronological age. (**B**) aTL and chronological age. (**C**) aTL and DNAmTL. Females and males distinguished by pink circles and blue triangles respectively.

Both measures exhibited small but significant negative correlations with chronological age despite the narrow age range of the sample, with DNAmTL showing stronger associations (aTL: r = −0.13, *p* = 0.040; DNAmTL: r = −0.25, *p* < 0.001) (Figure 2; Table 2A). These associations translated to an average decrease of 273 bp for each one-year increase in participant chronological age for the aTL measure, and 30 bp decrease for each one-year increase in participant chronological age for the DNAmTL measure. Correlations between aTL and DNAmTL were also weak (r = 0.20, *p* = 0.001), but remained relatively unchanged following adjustment for chronological age (Table 2B). Sensitivity analyses with additional control for blood cell proportions resulted in slightly increased correlations among all measures (Supplementary Table 1).

**Table 2:** Correlations among TL measures and chronological age before and after adjustment for chronological age. Statistic shown is Pearson correlation coefficient observed in partial correlation controlling for sex.

**2A**	**Raw Measures**	
	**Age**	**DNAmTL**
DNAmTL	−0.25^***^	
aTL	−0.13^*^	0.20^**^
**2B**	**Age-Adjusted Measures**	
	**Age**	**DNAmTL**
DNAmTL	0.00	
aTL	0.00	0.18^**^

(**A**) correlation among raw TL measures. (**B**) Correlation among age-adjusted TL measures. Age-adjusted performed by extracting residuals of each TL measure regressed onto chronological age independently in males and females. ^*^p < 0.05; ^**^p < 0.01; ^***^p < 0.001.

We also tested for differences in TL measurements between older and younger participants distinguished using a median split of the sample (μ_YOUNG_ = 10.13 years; μ_OLD_ = 12.63 years). DNAmTL estimates were significantly shorter in older participants (8.00 kb vs. 8.08 kb, *p* < 0.001). aTL estimates were also shorter in older participants, but this difference was not statistically significant (9.54 kb vs. 10.21 kb, *p* = 0.09).

### Associations between TL measures and external validity metrics

**Figure 3:**
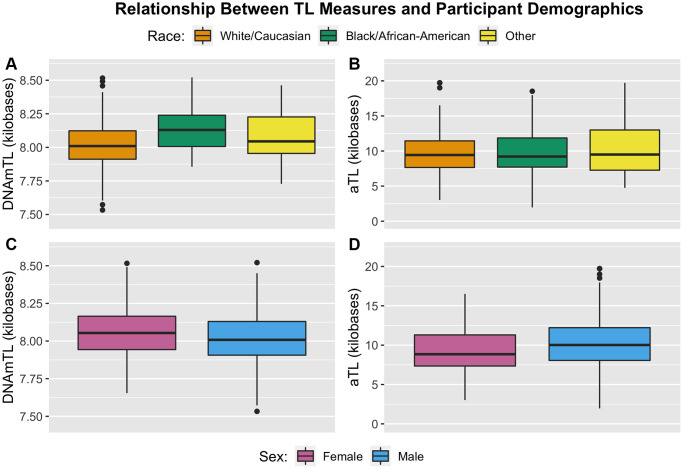
**Boxplots illustrating distribution of TL measures by participant demographic factors of race (top) and sex (bottom).** (**A**) DNAmTL partitioned by racial status. (**B**) aTL partitioned by racial status. (**C**) DNAmTL partitioned by sex. (**D**) aTL partitioned by sex.

Both TL measures exhibited significant differences in mean value as a function of sex (Figure 3; Table 3). For DNAmTL, males exhibited significantly shorter TL relative to females (8.02 kb vs. 8.06 kb, *p* = 0.029). For aTL measures, the opposite trend was observed, with males exhibiting significantly longer TL relative to females (aTL: 10.40 kb vs. 9.36 kb, *p* = 0.012). Differences in TL as a function of racial groups were observed for the DNAmTL measure, with those identifying as White exhibiting significantly shorter TL relative to those identifying as Black/African-American (8.01 kb vs. 8.13 kb, *p* = 2.30E-05). By contrast, no differences across racial strata were observed in aTL measurements (Figure 3). For both measures, no differences were observed as a function of ethnicity or maltreatment exposure. Full models with additional covariate adjustments for blood cell proportion and demographic factors resulted in similar findings (Supplementary Table 2).

**Table 3:** Results of generalized estimation equation models testing associations between TL measures and external validity metrics.

	**DNAmTL**			**aTL**		
	***β* [95% CI] *p*-value**			***β* [95% CI] *p*-value**		
Biological Sex (Males vs. Females)	**−0.27**	**[−0.51, −0.03]**	**0.029**	**0.32**	**[0.07, 0.56]**	**0.012**
Ethnicity (Hispanic vs. Non-Hispanic)	−0.15	[−0.43, 0.14]	0.320	−0.09	[−0.58, 0.41]	0.730
Race (Black/African-American vs. White)	**0.74**	**[0.44, 1.03]**	**<0.001**	−0.04	[−0.37, 0.29]	0.820
Race (Other vs. White)	0.28	[−0.09, 0.65]	0.140	0.17	[−0.21, 0.55]	0.370
Maltreatment (Exposed vs. Comparison)	−0.13	[−0.41, 0.14]	0.330	0.16	[−0.18, 0.51]	0.360

Coefficients reflect standard deviation difference in age-adjusted TL between groups. All models included covariate control for chronological age. Models testing for differences in TL as a function of ethnicity, race, and maltreatment status included additional covariate control for sex. All models included random effect for family ID to account for partial nesting of siblings within families. Significant results in bold. Abbreviation: CI: confidence interval.

### Exploratory associations between TL measures, pubertal stage and paternal age

We conducted exploratory analyses to test for associations between TL measures, pubertal development, and paternal age. Generalized estimating equation models revealed no associations between DNAmTL measurements and pubertal development measured using Tanner staging in both base and full models. Similarly, aTL measurements were not associated with pubertal development (Table 4A). Paternal age at birth was also not associated with either TL measure in base and full models (Table 4B).

**Table 4:** Results of generalized estimating equation models testing associations between TL measures and exploratory metrics.

**4A**	**Tanner Stage: Base Models**			**Tanner Stage: Full Models**		
	***β* [95% CI] *p*-value**			***β* [95% CI] *p*-value**		
DNAmTL	**−**0.04	[**−**0.20, 0.11]	0.590	**−**0.11	[**−**0.24, 0.02]	0.111
aTL	0.15	[0.00, 0.30]	0.051	0.14	[**−**0.03, 0.30]	0.100
**4B**	**Paternal Age: Base Models**			**Paternal Age: Full Models**		
	***β* [95% CI] *p*-value**			***β* [95% CI] *p*-value**		
DNAmTL	0.01	[**−**0.01, 0.03]	0.240	0.01	[**−**0.01, 0.02]	0.408
aTL	0.00	[**−**0.01, 0.02]	0.710	0.01	[**−**0.01, 0.02]	0.510

Age-adjusted TL measures were standardized within sex for analysis. Base models included covariate control for chronological age and sex. Full models included additional covariate control for blood cell proportions, BMI, income, race, and ethnicity. All models included random effect for family ID to account for partial nesting of siblings within families. (**A**) Models predicting measures of TL by Tanner stage. Coefficients reflect SD increase in age-adjusted TL for each one unit increase in Tanner stage. (**B**) Results of models predicting measures of TL by paternal age. Coefficients reflect SD increase in age-adjusted TL for each one-year increase in paternal age. Abbreviation: CI: confidence interval.

## DISCUSSION

We assessed the comparability of qPCR (aTL) and DNA methylation-based (DNAmTL) measures of TL in a high-risk pediatric cohort. Both measures exhibited expected decreases in TL with advanced chronological age. However, only DNAmTL replicated expected associations across external validity metrics, showing significantly shorter TL in males relative to females and White relative to Black/African-Americans. By contrast, aTL measures exhibited significant sex differences, but in the opposite direction from expectations.

The observed correlation between DNAmTL and aTL measures was positive and small (r = 0.20, *p* = 0.001), but within the range of values previously reported for studies investigating concordance between DNAmTL and qPCR-based measures (range= −0.01–0.41) [32, 34]. Notably, the previously reported correlation coefficients tended to be lower within datasets with the smallest age range (r = −0.01, age range = 3 years; r = 0.08, age range = 4 years), whereas datasets with a larger age range tended to be characterized by correlations of a higher magnitude (r = 0.38, age range = 57 years; r = 0.41, age range = 41 years) [32, 34]. Given the 5-year age range in the current study, the observed correlation between aTL and DNAmTL is of an expected magnitude given previously reported values.

Average TL for the analytical sample varied significantly between measures estimated using qPCR and those estimated using DNA methylation, with aTL estimates tending to be larger than those generated using DNAmTL. Despite differences between measures, observed sample means for both (aTL: 9.88 kb; DNAmTL: 8.04 kb) were within previously reported estimates generated by Southern blot, which tend to range between 7 kb to 10 kb for adolescents [30, 41]. Both measures were also significantly correlated with chronological age, although to a smaller degree than in previous reports. A large-scale meta-analysis including over 100 studies reported a pooled age correlation of r = −0.29 for qPCR measures [42], and estimates for DNAmTL tend to be -0.60 or greater in magnitude [32, 34]. The modest correlations observed here may be a function of the relatively narrow age range for the current sample, which limits our ability to disentangle variability due to chronological age from inter-individual variability at any given age. Even so, the observed decrease of 30 bp per one-year increase in participant chronological age for DNAmTL estimates is similar to previous studies reporting decreases of 18 and 21 bp per year [32, 34], and is within 20–60 bp range typically observed for TL estimates generated using Southern blot and/or qPCR [2, 29–31]. By contrast, the 273 bp per year decrease estimated for aTL estimates was much larger than previous reports.

Previous work has highlighted limitations in the estimation of absolute TL in kilobases from qPCR measurements using conversion equations based on subsets of samples measured with more precise methods such as Southern Blot or flow FISH [43]. These limitations may be attributable to differences in the biochemical processes utilized in qPCR (i.e., exponentiation) relative to flow FISH or Southern Blot (i.e., hybridization), resulting in imprecise estimates for values at tails of the distribution when the relationship between methods is forcibly modeled linearly. For example, one should not expect accurate estimation of absolute TL in kilobases when the R^2^ for the correlation between T/S ratios and flow FISH TL is modest (e.g., R^2^ = 0.56). By contrast, the approach to generate aTL values in the current work relies only on qPCR data using double-stranded oligomer standards. This approach is advantageous in that the biochemical process by which standard concentrations are estimated is the same process by which samples are estimated, resulting in a significantly higher correlation between T/S ratio values and aTL estimates (R^2^ = 0.98). However, this approach is not without limitations. Specifically, the approach is challenged by the difficulty in accurately constructing oligomer standards with very low DNA concentrations. Slight deviations between expected and actual concentrations for these standards can result in under or overestimated aTL values, which may have occurred here.

Exploratory analyses failed to replicate hypothesized associations between TL measures and paternal age. Previous work, including a meta-analysis of nearly 20,000 participants, has shown evidence for a paternal age effect on TL in adulthood, where advanced paternal age is associated with longer TL in offspring [31, 39, 44]. Paternal age was also associated with salivary TL measured via the T/S ratio in a cohort of children from New Zealand [38], but analyses were conducted without controls for age or sex. Therefore, it is possible that differences in TL resulting from paternal age do not manifest in leukocytes until later in life, or that associations between TL and paternal age are obscured by sex and age effects. We were also unable to distinguish differences in TL across pubertal development. The co-occurrence of accelerated pubertal development with accelerated biological aging has been contextualized as an evolutionary adaptation to early-life adversity, wherein reproductive fidelity is prioritized at the cost of long-term survival to maximize the possibility of reproduction in a high stress environment [40]. Our work provides mixed support for this hypothesis. Individuals who were investigated for child maltreatment were characterized as having significantly advanced Tanner staging in the absence of any differences in chronological age. However, age-adjusted TL was not significantly associated with advanced pubertal development, irrespective if it was assessed via qPCR or DNA methylation. It may be that advanced pubertal development precedes changes in biological aging, and we intend to continue this analysis as the cohort expands with future waves of data collection and larger sample sizes.

Maltreatment exposure was not associated with accelerated biological aging in DNAmTL or aTL measurements. Features of the study design, such as how maltreatment was assessed, may have impacted our ability to detect these associations. A meta-analysis including 41 studies identified significant variation in the strength of associations with TL based on how adversity was evaluated [45]. Specifically, studies with a narrow focus on abuse and/or neglect tended to report lower effect sizes relative to studies with more comprehensive adversity assessment. Data collection for the CHS is still ongoing, and as a result we conducted analyses within a subset of the final cohort using both a dichotomous child maltreatment variable (investigated for maltreatment vs. comparison). As the remainder of the CHS cohort is assembled more detailed information is being collected from county level records for all participants, and future work with this cohort will be able to include more nuanced maltreatment variables. This is especially relevant for the DNA methylation-based indicator DNAmTL given recent evidence for the increased sensitivity of epigenetic aging measures to experiences of threat and violence relative to other domains of early adversity [46, 47].

We acknowledge limitations within the current study. Participant recruitment for the CHS is still ongoing, and a limited sample size for demographically matched comparison children may have also detracted from our ability to detect maltreatment effects in main analyses. Future analyses within the larger cohort will include more detailed maltreatment variables such as timing, duration, and severity of maltreatment, which may contribute to associations between early adversity and measures of cellular aging [45, 48]. A lack of agreement between aTL and DNAmTL measures could also be attributed to processing differences in tissue source. Epigenetic data for DNAmTL was based on DNA extracted from whole blood using a magnetic bead protocol. By contrast, aTL measurements were generated on DNA extracted from buffy coat using a salting-out method. Although buffy coat cells make up the preponderance of leukocyte cells in whole blood, it remains possible that differences in the distribution of leukocyte cells or DNA extraction protocols could have contributed to differences between the two measures. The current findings are also limited by their cross-sectional nature. It remains contentious as to whether static biological age or the rate of change in biological age across time is a better predictor of health and longevity [49–51]. Even so, inter-individual differences in TL established in early life may ‘set the trajectory’ for between-person differences across the lifespan [52]. Thus, it is uncertain whether the rate of telomere shortening or static TL better forecasts the future health of adolescents. The CHS offers an intriguing avenue to test these and other hypotheses related to the concordance among aTL and DNAmTL measures as future waves of data collection continue.

Overall, our findings highlight important limitations of high-throughput based measures of TL when applied within a pediatric cohort. In most instances, DNAmTL replicated associations with external validity metrics or showed effect sizes in the hypothesized direction. By contrast, aTL measurements were positively skewed and tended to exhibit relationships with few external validity measures. In sum, our findings extend previous research in adults and provide support for the utility of DNAmTL as a marker of biological aging for future research in pediatric populations.

## METHODS

### Study design and sample recruitment

Participants were drawn from the ongoing CHS, a large multidisciplinary study designed to provide prospective, longitudinal data on the health and development of children with and without a history of maltreatment. The CHS is recruiting a large state-wide cohort of children exposed to maltreatment within the past 12 months, defined here as investigated reports of neglect, physical abuse, or sexual abuse, and demographically matched non-maltreated comparison children aged 8–13 [35]. The goal of the CHS is to elucidate the multiple etiological processes believed to play a role in the onset and maintenance of adverse health outcomes among victims to better inform intervention research and reveal opportunities for reversibility. The Pennsylvania State University Institutional Review Board approved the study, and informed assent (child) and consent (caregiver) was obtained from all participants.

Children with a recent (<12 months) report of maltreatment exposure were identified through Pennsylvania’s Statewide Child Welfare Information System (CWIS). Subjects with recent involvement in the CWIS were invited to participate in the study through home mailings and phone contact by study coordinators. Eligibility for participation included: (1) aged 8 to 13 years, (2) subject of a CWIS maltreatment report (i.e., an allegation is made and investigated) within the past 12 months, and (3) agreement of participation by a non-abusing caregiver. Non-maltreated comparison children are recruited via targeted advertisements from the same Pennsylvania counties as maltreated children with the goal of demographically matching at least one maltreated child based on age, race, ethnicity, sex, income level, and region within the State. Eligibility for participation includes: (1) no previous CWIS reports (i.e., via screening through CWIS prior to enrollment), and (2) demographic match to a maltreatment participant.

Cross-sectional data for the current study is drawn from Time 1 (baseline) assessment of currently enrolled CHS participants. Of the 439 participants who have completed Time 1, 401 consented to and successfully completed blood draws (the 38 missing blood samples included: 1 caregiver refusal, 33 participant refusals, and 4 attempted but incomplete blood draws). Of the 401 currently consented participants, 286 samples were available at the time of DNA methylation analyses, with 269 samples surviving DNA methylation *and* TL quality control metrics. Summary statistics for these participants are provided in Table 1. We tested for differences between those missing DNA methylation and/or TL data (*N* = 170) and those with data for both measures (*N* = 269). In these analyses, the sample with incomplete data had significantly younger paternal age at birth relative to the analytical sample (27.63 vs. 29.32, *p* = 0.02). No significant differences were detected for remaining demographic or covariate measures.

### Assessment of DNA methylation and calculation of DNAmTL

During participants’ visit, blood was collected by professional phlebotomists via venipuncture into two 10 mL EDTA tubes. A small volume of whole blood (3 mL) was then aliquoted into a 4mL EDTA tube and stored at −80^o^C before DNA extraction and DNA methylation assays. DNA for methylation assays was extracted from whole blood using QIAsymphony (Qiagen, Germany). Epigenetic methylation assays were conducted using the Infinium methylation EPIC array (Illumina), which quantifies the methylation status of over 850,000 CpG and non-CpG sites. Resulting methylation measures were used to calculate DNAmTL according to published methods [32]. Briefly, the DNAmTL measure was developed by regressing leukocyte TL measured using Southern Blot onto blood methylation levels, and subsequently using elastic net regression to extract a final set of 140 CpGs forming a predictive model. We extracted this set of 140 CpGs and applied published regression coefficients to calculate DNAmTL measurements for participants in our analytical sample. Full details on sample processing and quality control for methylation analyses are provided in Supplementary Methods.

### Assessment of telomere length via qPCR and aTL calculation

TL measurements generated using qPCR were determined based on DNA extracted from buffy coat cells. Buffy coat cells were isolated using centrifugation to separate plasma followed by treatment with 0.5× red blood cell lysis buffer (Invitrogen). Buffy coat cells were stored at −80^o^C prior to DNA extraction using Gentra Puregene kits (Qiagen) with no modification from factory guidelines. DNA concentration was determined using Quant-iT PicoGreen Reagent (Qiagen). DNA purity and quality were assessed using 260/230 and 260/280 ratios for all samples (mean_260/230_ = 1.06; mean_260/280_ = 1.93). An additional subset of samples (*N* = 30; 11.1%) were evaluated using the Agilent 2200 TapeStation to determine the DNA Integrity Number (DIN) with mean_DIN_ = 8.6, indicating intact, minimally degraded DNA.

TL assays were conducted following a qPCR method originally developed by O’Callaghan and Fenech [17] using a Rotor-Gene Q thermocycler connected to an uninterruptible power source (CyberPower), which has been shown to decrease variability in TL measured via qPCR [53]. Each qPCR assay was comprised of two runs, one quantifying telomere content (T), and a second run quantifying genome copy number (S) using the single copy gene *IFNB1*. The two runs (T & S) were always performed on the same day using the same DNA aliquot, which was stored at 4°C between runs (~2.5 hours).

Raw fluorescence data was extracted from Rotor-Gene Q software for post-processing using LinRegPCR [54]. Within LinRegPCR, individual windows of linearity were established for standards and analytical samples to estimate baseline DNA content (N_0_), Cq values, and amplification efficiency per amplicon (T or S) [55]. For aTL calculations, a conversion factor was generated as the average ratio of N_0_ estimates to expected concentration of the oligomer standards. N_0_ estimates for analytical samples were then divided by this conversion factor to estimate kb telomeric DNA and genome copy number for each sample, which were used for final calculation of aTL as:


$$aTL=\frac{Estimated\;kb\;Telomeric\;DNA}{Estimated\;Genome\;Copy\;Number\times 92}$$

To control for inter-assay variability, 3 control samples were assessed on each T run and each S run. The average inter-assay CV for control sample aTL estimates was 14.0%. A random selection of 21 samples was reassessed for explicit purposes of calculating the interclass correlation coefficient (ICC), an indicator of measurement reliability. The resulting ICC for aTL estimates was 0.586, indicating moderate reliability. Full details on qPCR assays for telomere length, including reaction mix composition and sequences for primers and standards, are summarized in Supplementary Table 3 in accordance with guidelines recommended by the Telomere Research Network (https://doi.org/10.31219/osf.io/9pzst).

### Pubertal development

Pubertal development was assessed using Tanner staging, which indexes the development of physical traits on a five point scale ranging from 1 (prepubertal) through 5 (fully mature) [56–58]. Each participant rated their stage of pubic hair development and breast (females only) or testis (males only) development. The final pubertal development measure was calculated as the average across these two separate ratings.

### Other measures

Chronological age, sex, race, ethnicity, and BMI were included as covariates due to known associations with TL. Sex was determined via self-report and cross-validated using DNA methylation predicted sex. Two participants self-identified as ‘other/transgender’ but had not undergone any gender-reassignment treatment and were therefore coded as their cross-validated sex. Race was coded as ‘White’, ‘Black/African American’, or ‘Other’ (American Indian, Alaskan Native, Multiracial, or Other) based on reports provided by caregivers. Ethnicity was coded as either ‘Hispanic’ or Non-Hispanic’. BMI was measured as the total body mass in kilograms divided by the squared body height in meters. Family income was self-reported by caregivers as current total household income before taxes in increments of $10,000 (e.g., under $10,000 coded as ‘0’, $10,000–$19,999 coded as ‘1’, $20,000–$29,999 coded as ‘2’ and an income over $120,000 coded as ‘11’). Differences as a function of maltreatment exposure were tested using a dichotomous variable that distinguished between those with CWIS maltreatment reports and those with no CWIS reports, i.e., maltreatment (*N* = 222) vs. comparison (*N* = 47). Paternal age at birth was determined using date of birth for the biological father and child where known. In instances where paternal date of birth was unknown (*N* = 11), it was approximated by subtracting the child’s current age from the estimated current age of the biological father as reported by the current guardian.

### Statistical analyses

Statistical analyses were performed using R Studio V 4.0.2. Tests for mean differences in demographic variables between maltreatment and comparison groups were assessed using two-tailed *t*-tests for continuous variables and two-way Chi-Square tests for categorical variables. We assessed continuous variables for skewness and kurtosis. Due to a subset of outlier measurements with values greater than 3 standard deviations above the mean (*n* = 7), aTL measurements violated assumptions of normality (skew_aTL_pre_ = 3.25, kurtosis_aTL_pre_ = 16.90). These 7 samples (2.6% of the analytical sample) were winsorized at 3% of the upper tail prior to analyses, after which aTL measurements were approximately normal (skew_aTL_post_ = 0.72, kurtosis_aTL_post_ = 0.58).

In order to compare measurement methods expressing TL in kb units, the current analyses utilized aTL measures generated using qPCR instead of the more commonly employed T/S ratio. Measurements of TL expressed using the T/S ratio and aTL were highly correlated with one another (r = 0.96, *p* < 0.001), and exhibited similar associations across external validity and exploratory metrics. As a result, only analyses with aTL are shown here. Measurement agreement between aTL and DNAmTL was tested using Bland Altman analysis [59]. Measurement bias is estimated as the mean difference between the two methods, with a zero-line indicating perfect agreement. Limits of agreement are calculated as the area within two standard deviations of the mean difference.

Tests for differences in DNAmTL and aTL were conducted using versions of each measure adjusted for chronological age. Adjustment was performed by extracted non-standardized residuals of each measure regressed onto chronological age. To account for sex differences in TL, age-adjustment was performed independently in males and females. In instances when data for a given factor was unavailable for the full sample (i.e., Tanner stage and paternal age) age-adjustment was performed within the subsample with complete data for that factor (N_Tanner_ = 265; N_Paternal_ = 223). These residuals were then standardized for use in generalized estimating equations to compute standardized effect sizes and *p*-values reported in text and tables. Group means in kilobases are provided to enhance interpretability of results. Tests for differences in age-adjusted DNAmTL and aTL were tested using generalized estimating equations (function *geeglm*). Base models included covariate adjustment for sex and chronological age only. Full models included additional covariate control for blood cell proportions estimated from methylation data using an established reference-based approach [60], as well as BMI, family income, race, and ethnicity. To account for partial nesting of siblings within families (4 families with three siblings, 3 families with two siblings, and 185 families with a single child), all models were estimated with standard errors clustered at the family level with family ID as the repeated factor.

Within our analytical sample, 4 individuals were missing covariate data for family income. No significant differences in other covariates were observed between these individuals and the remaining sample, and therefore missing data were addressed using multiple imputation and complete case analysis. We created 5 imputed datasets using IVEWare [61] and replaced missing values with the average of imputed values across these 5 iterations. Missing values for family income were imputed using all demographic variables.

## Supplementary Materials

Supplementary Methods

Supplementary Tables
