# Supplementary material for: Comparing qPCR and DNA methylation-based measurements of telomere length in a high-risk pediatric cohort
Source: Aging (Albany NY). 2022 Jan 24;14(2):660–77. doi: 10.18632/aging.203849 (PMC8833135; doi:10.18632/aging.203849)
Supplement: Supplementary Methods [file aging-14-203849-s001.pdf]

SUPPLEMENTARY METHODS FOR DNA METHILATION

DNA extraction

Genomic DNA was isolated from whole blood samples using a semi-automated approach (Qiasymphony, Qiagen). Genomic DNA purity and concentration was assessed using a nanophotometer (ImplenP300, Implen).

DNA methylation analyses

We used the Infinium methylation EPIC Beadchip (EPIC array, Illumina, San Diego CA, USA) to describe variation in DNA methylation across the genome. Genomic DNA (1ug) from whole blood was treated with sodium bisulfite using the Zymo EZ-96 DNA Methylation Kit™ (Zymo Research, Orange, CA, USA) with 200ng of bisulfite-treated DNA amplified, fragmented, and hybridized on the EPIC array. Samples were randomized across plates to avoid potential confounding between sources of technical variation and phenotypes of interest (e.g., maltreatment status). The resulting raw intensity values (idat files) are directly loaded into R for quality control and normalization using the Meffil package [1]. We used normal-exponential out-of-band (noob) for background correction and dye-bias adjustment. Samples and probes with low signal intensity were removed. Concordance between predicted biological sex based on DNA methylation data and self-reported gender were verified for each sample with discordant samples removed. Finally, we used a Bayes method (ComBat) to correct for sources of technical variation (i.e., slide) [2].

Blood cell types

Between samples differences in blood cell proportions were estimated using an established reference-based approach and included as covariates as needed for certain robustness checks [3].

REFERENCES

1. Min JL, Hemani G, Davey Smith G, Relton C, Suderman M. Meffil: efficient normalization and analysis of very large DNA methylation datasets. Bioinformatics. 2018; 34:3983–89. https://doi.org/10.1093/bioinformatics/bty476 PMID:29931280
2. Johnson WE, Li C, Rabinovic A. Adjusting batch effects in microarray expression data using empirical Bayes methods. Biostatistics. 2007; 8:118–27. https://doi.org/10.1093/biostatistics/kxj037 PMID:16632515
3. Houseman EA, Accomando WP, Koestler DC, Christensen BC, Marsit CJ, Nelson HH, Wiencke JK, Kelsey KT. DNA methylation arrays as surrogate measures of cell mixture distribution. BMC Bioinformatics. 2012; 13:86. https://doi.org/10.1186/1471-2105-13-86 PMID:22568884
