# Supplementary material for: Comparing qPCR and DNA methylation-based measurements of telomere length in a high-risk pediatric cohort
Source: Aging (Albany NY). 2022 Jan 24;14(2):660–77. doi: 10.18632/aging.203849 (PMC8833135; doi:10.18632/aging.203849)
Supplement: Supplementary Tables [file aging-14-203849-s002.pdf]

SUPPLEMENTARY TABLES

Supplementary Table 1. Partial correlations among TL measures and chronological age with additional control for blood cell proportions.

**A**	**Raw measures**	
	**Age**	**DNAmTL**
DNAmTL	−0.26***	
aTL	−0.13*	0.21***
**B**	**Age-adjusted measures**	
	**Age**	**DNAmTL**
DNAmTL	0.05	
aTL	0.00	0.18**

Partial correlations included control for sex as in main text with additional adjustment for lymphocyte, monocyte, and granulocyte proportions. Statistic shown is Pearson correlation coefficient. (**A**) Raw measures. (**B**) Age-adjusted measures. Age-adjusted performed by extracting residuals of each TL measure regressed onto chronological age independently in males and females. *$p < 0.05$; **$p < 0.01$; ***$p < 0.001$.

Supplementary Table 2. Results of fully adjusted generalized estimation equation models testing associations between TL and external validity metrics.

	**DNAmTL**			**aTL**		
	***β* [95% CI] *p*-value**			***β* [95% CI] *p*-value**		
Biological Sex (Females vs. Males)	**−0.35**	**[−0.52, −0.12]**	**0.002**	**0.34**	**[0.08, 0.60]**	**0.010**
Ethnicity (Hispanic vs. Non-Hispanic)	−0.21	[−0.48, 0.06]	0.123	−0.17	[−0.67, 0.32]	0.492
Race (Black/African-American vs. White)	**0.51**	**[0.24, 0.77]**	**<0.001**	−0.17	[−0.53, 0.19]	0.354
Race (Other vs. White)	0.12	[−0.20, 0.44]	0.454	0.12	[−0.26, 0.50]	0.547
Maltreatment (Exposed vs. Comparison)	−0.19	[−0.43, 0.05]	0.115	0.12	[−0.26, 0.50]	0.549

Coefficients reflect SD difference in age-adjusted TL between groups. All models included covariate control for chronological age, BMI, income, blood cell proportions, as well as sex, race, and ethnicity. All models included random effect for family ID to account for partial nesting of siblings within families.

Supplementary Table 3. TRN reporting guidelines.

Item	Description
**Sample type, storage, extraction, and integrity**	
Sample type	DNA samples were extracted from buffy coat cells separated from whole blood collected in 10mL EDTA tubes. Buffy coat cells were isolated using centrifugation to separate plasma followed by treatment with 0.5× red blood cell lysis buffer (Invitrogen).
Sample storage conditions	Buffy coat cells were stored at -80°C in a solution buffer comprised of phosphate buffered saline pH 7.2 + EDTA (2mMol) + bovine serum albumin (0.5%) prior to extraction. Duration between sample collection and DNA extraction ranged from 29 days to 2.17 years (mean = 6.84 months; SD = 4.80 months).
DNA extraction method	GentraPuregene (Qiagen) with no modification from factory guidelines.
DNA storage conditions, including freeze-thaw cycles	DNA was stored −80°C in Qiagen DNA Hydration Solution. On average there were three freeze thaws for DNA samples between extraction and the qPCR assay. The first thaw was done to determine DNA concentration using Quant-iTPicoGreen reagent (Qiagen). PicoGreen assays occurred in two batches. The first batch occurred between 11/18/2019 and 12/23/2019 with an average duration of 7.68 months between DNA extraction and the PicoGreen assays. The second batch of PicoGreen assays occurred between 8/21/2020 and 8/26/2020 with an average duration of 0.97 months between extraction and PicoGreen assays.<br><br>A second freeze thaw was needed to perform a dilution for the qPCR assay. The final thaw occurred when the sample was assayed. Samples needing to be reassessed on qPCR assays (*n* = 57; 21.1%) were thawed one additional time. DNA samples from the first batch of PicoGreen assays were stored for an average of 13.13months between the PicoGreen assay and qPCR assay. DNA samples from the second batch of PicoGreen assays were stored for an average of 4.52 months between the PicoGreen assay and the qPCR assay.
Method of documenting DNA quality and integrity	DNA was quantified for all samples using Quant-iTPicoGreen Reagent (mean = 51.18 ng/uL). DNA purity and quality was assessed using 260/230 and 260/280 ratios for all samples. ($mean_{260/230} = 1.06$; $mean_{260/280} = 1.93$). An additional subset of samples (*n* = 30, 11.1%) were evaluated using the DNA Integrity Number generated by the Agilent 2200 TapeStation with $mean_{DIN} = 8.6$, indicating intact, minimally degraded DNA. No exclusionary criteria was imposed prior to assays.
Percentage of samples specifically tested for DNA quality and integrity	All samples were subjected to quality control via evaluation of 260/280 and 260/230 ratios. A subset of samples (30/270 = 11.1%) were subjected to quality assessment via TapeStation.
**qPCR assay**	
Method (qPCR, MMqPCR, aTL, etc.)	qPCR assays to calculate absolute telomere length (aTL) were structured such that each assay comprised two qPCR runs, one run quantifying telomere content in kilobases (T) and a second run quantifying genome copy number (S) using the single copy gene *IFNB1*. The two runs (T & S) were always performed on the same day using the same DNA aliquot which was stored at 4°C between runs (~2.5 hours). Each run hosted triplicate reactions of 21 samples, 6 standards, 3 positive controls, and 1 no template control on 100 well disks.<br><br>A total of 20 qPCR assays were performed across a period of 31 days from 12/16/2020 to 1/16/2021 for analysis of all samples.
PCR machine type	Qiagen Rotor-Gene Q using 100 well disks
Source of master mix and reagents, and final reaction volume	The final reaction mix for the telomeric and *IFNB1* reactions contains 1x QuantiTectSYBR Green Master Mix (Qiagen), 0.2U Uracil Glycosylase (Thermo Fisher Scientific), 0.1 uM forward primer, 0.1 uM reverse primer, and 6 ng DNA in a 20 uL reaction.<br><br>Primers are purchased from IDT in lab-ready format (HPLC purified, 100uM in IDTE Buffer pH 8.0)
Telomere primer sequences and concentration<br>Single copy gene name, primer sequences, and concentration	Forward Primer: 5'-CGGTTTGTTTGGGTTTGGGTTTGGGTTTGGGTTTGGGTT-3′<br>Reverse Primer: 5'-GGCTTGCCTTACCCTTACCCTTACCCTTACCCTTACCCT-3′<br>*IFNB1* Forward Primer:5′-TGGCACAACAGGTAGTAGGCGACAC-3′<br>*IFNB1* Reverse Primer:5′-GCACAACAGGAGAGCAATTTGGAGGA-3′
Full PCR program description including temperature, times, and cycle numbers	50°C – 2 min<br>95°C – 15 min<br>95°C – 15 s<br>60°C for 1 min<br>72°C for 30 sec (data acquisition)<br>} 40 cycles<br>Melt 60°C to 99°C rising 1°C per step with 5 sec per step
PCR efficiency of single copy gene and telomere primers	<u>Estimates from RotorGene</u><br>Telo: R^2 = 0.999102 (range 0.99401–0.9987; Efficiency = 2.0465 (range 2.00–2.09)<br>IFNB1: R^2 = 0.999319 (range 0.99793–0.99991); Efficiency = 1.987 (range 1.95–2.07)<br><u>Estimates from LinRegPCR</u><br>Telo Amplicon Efficiency = 1.890<br>IFNB1 Amplicon Efficiency = 1.847
Source and concentration of control samples and standard curve	3 positive controls were randomly selected from within the sample to control for variation across T and S runs. Standards consisted of double stranded oligomers purchased from IDT as lyophilized pellet with PAGE purification.<br><br>Standard curves for T runs consisted of 84 bp double stranded oligomer comprised of 16 copies of canonical telomere repeat. Telomere Standard A had concentration 0.15 ng/uL, which equates to 5.86e + 08 kb telomeric DNA when 4uL is used in the qPCR assay. A series of 1/10 serial dilutions were performed to generate a total of 6 standards for each T run comprising a range of 5.86e + 08 to 5.86e + 5.86e + 03 kb telomeric DNA.<br><br>Standard curves for S runs consisted of 83 bp double stranded oligomer corresponding to the region of IFNB1 genomic DNA flanked by IFNB1 primers. IFNB1 Standard 1 had concentration 0.0005 ng/uL, which equates to 1.18e + 07 diploid genomes when 4uL is used in the qPCR assay. A series of 1/10 serial dilutions were performed to generate a total of 6 standards for each S run comprising a range of 1.18e+07 to 1.18e + 02 diploid genome copies.
Telomere Standard Oligomer Sequences	Sense: 5′-CCC TAA CCC TAA CCC TAA CCC TAA CCC TAA CCC TAA CCC TAA CCC TAA CCC TAA CCC TAA CCC TAA CCC TAA CCC TAA CCC TAA CCC TAA-3′<br><br>Anti-sense: 5′-TTA GGG TTA GGG TTA GGG TTA GGG TTA GGG TTA GGG TTA GGG TTA GGG TTA GGG TTA GGG TTA GGG TTA GGG TTA GGG TTA GGG TTA GGG-3′

IFNB1 Standard Oligomer Sequences	Sense: 5′-GCACAACAGGAGAGCAATTTGGAGGAGACACTTGTTGGTCATGTTGACAACACGAACAGTGTCGCCTACTACCTGTTGTGCCA-3′<br>5′-TGGCACAACAGGTAGTAGGCGACACTGTTCGTGTTGTCAACATGACCAACAAGTGTCTCCTCCAAATTGCTCTCCTGTTGTGC-3′
**Data analysis**	
Mean and standard deviation or median range of telomere lengths	aTL mean (SD) = 10.17 kb (4.78kb)
Number of sample replicates	Each sample was assessed for T and S on a single run with three replicates within the run. If the sample did not pass quality control criteria described below it was run a second time.
Level of independence of replicates	Replicates were drawn from the same DNA aliquot (i.e., the same tube).
Analytic method, considering replicate measurements, to determine final length	Raw fluorescence data was extracted from RotorGene Q software for post-processing using LinRegPCR. Within LinReg, individual windows of linearity were established for standards and analytical samples to estimate baseline DNA content (N_0) and Cq values. Control reactions targeting genome copy number were treated an independent amplicon groups on T runs. Similarly, control reactions targeting telomeric content were treated as independent amplicon groups on S runs. Following processing, N_0, Cq, and efficiency values were extracted for calculation of aTL using the formula below.<br>$$aTL = \frac{Estimated\ kb\ Telomeric\ DNA}{Estimated\ Genome\ Copy\ Number \times 92}$$<br>For aTL calculations, a conversion factor was generated as the average ratio of baseline DNA content estimated by LinReg (N_0) to expected concentration of the oligomer standards across all replicates of all standards, excepting any replicates flagged as aberrant by LinReg. N_0 estimates for analytical samples were then divided by this conversion factor to calculate kb telomeric DNA estimates and genome copy number estimates for each replicate on a given run. When applicable, baseline estimates for the no template control were subtracted from estimates of the analytical samples prior to applying the conversion factor. The average kb telomeric DNA estimates and genome copy number estimates across replicates were used to calculate aTL values.
Method of accounting for variation between replicates	Replicates flagged as noisy of having baseline errors by LinReg were dropped prior to any calculations. On average 2.75 T replicates and 2.95 S replicates (*excepting the no-template control*) were flagged by LinReg per run. When the coefficient of variation across triplicate estimates of telomere content or genome copy number was greater than 15%, replicate estimates were evaluated based upon their deviation from mean across triplicates. If one replicate deviated from the mean by more than 15% it was considered an outlier and the mean was recalculated using two replicates. Excepting samples that were rerun, an average of 10.9 T replicates and 7.3 S replicates were dropped per run (*in this case aTL values were calculated using the average across duplicate measures*).<br>In the case where coefficient of variation across replicates was still greater than 15% after removal of a single outlier, or was greater than 15% without a clear outlier defined by the criteria above, the sample was reassessed for both telomere content and genome copy number, and subjected to the same quality control evaluation. A total of 67 (20.6%) samples were rerun a second time.
Method of accounting for well position effects within plates	The unique rotary design of the Rotor Gene Q is optimized to minimize well position effects. As such no accounting for well position effects was performed.
Method of accounting for between plate effects	To control for inter-assay variability, the telomeric content and genome copy number were assessed for three control samples on each T run and each S run. For each run, the estimated baselines (N_0) for control reactions targeting telomeric content and genome copy number were divided by the average estimated baselines across all runs to get a normalizing factor for that sample on a given run. This was done for all controls to get an average normalizing factor for that run. Baseline values for the standards and analytical samples were then divided by the normalization factor for a given run prior to calculating conversion factors and kb telomeric DNA estimates and genome copy number estimates. In this manner the average intra-run CV across replicate kb telomeric DNA estimates and genome copy number estimates was 5.64% and 5.76% respectively. The average inter-run CV across control kb telomeric DNA estimates and genome copy number estimates was 11.3% and 10.6% respectively. Inter-assay CV for resulting aTL estimates was 14.0% on average across the three control samples.
% of samples repeated and % of samples failing QC and excluding from further analyses	57/270 = 21.1% of samples repeated<br>1/270 = 0.4% of samples failed QC and excluded from analyses.
Acceptable range of PCR efficiency for single copy gene and telomere primers	1.80–2.00 (*10% variation*)
ICCs of samples/study groups to address variability	A random selection of samples (*n* = 21; 6%) were reassessed for the explicit purposes of calculating the ICC. This plate was subject to the same control for within and between plate variation as described above. ICCs were calculated at the level of aTL using a 2-way mixed effects model with a single measurement, i.e., ICC(A,1). General formulas and estimated values for these ICCs are provided below. (MS= mean square)<br>$$ICC(A,1) = \frac{MS_{Samples} - MS_{Error}}{MS_{Samples} + MS_{Error} + \frac{MS_{Assay} - MS_{Error}}{21}}$$<br>ICC = 0.586<br>ICC calculated with covariate adjustment for chronological age showed were slightly smaller in magnitude.<br>ICC_{Age} = 0.570
T/S ratio transformed to a z-score prior before comparison across methods/studies	N/A. No comparison across studies was conducted.
How samples nested within families were accounted for	Samples from the same family (siblings) were always run on the same plate.
